# Survey of CAM interest, self-care, and satisfaction with health care for type 2 diabetes at group health cooperative

**DOI:** 10.1186/1472-6882-11-121

**Published:** 2011-12-01

**Authors:** Ryan Bradley, Karen J Sherman, Sheryl Catz, Carlo Calabrese, Luesa Jordan, Lou Grothaus, Dan C Cherkin

**Affiliations:** 1 Bastyr University Research Institute (BURI), Kenmore, WA, USA; 2 Group Health Research Institute (GHRI), Seattle, WA, USA

## Abstract

**Background:**

Very little research has explored the factors that influence interest in complementary and alternative medicine (CAM) treatments. We surveyed persons with sub-optimally controlled type 2 diabetes to evaluate potential relationships between interest in complementary and alternative medicine (CAM) treatments, current self-care practices, motivation to improve self-care practices and satisfaction with current health care for diabetes.

**Methods:**

321 patients from a large integrated healthcare system with type 2 diabetes, who were not using insulin and had hemoglobin A1c values between 7.5-9.5%, were telephoned between 2009-2010 and asked about their self-care behaviors, motivation to change, satisfaction with current health care and interest in trying naturopathic (ND) care for their diabetes. Responses from patients most interested in trying ND care were compared with those from patients with less interest.

**Results:**

219 (68.5%) patients completed the survey. Nearly half (48%) stated they would be very likely to try ND care for their diabetes if covered by their insurance. Interest in trying ND care was not related to patient demographics, health history, clinical status, or self-care behaviors. Patients with greater interest in trying ND care rated their current healthcare as less effective for controlling their blood sugar (mean response 5.9 +/- 1.9 vs. 6.6 +/- 1.5, p = 0.003), and were more determined to succeed in self-care (p = 0.007). Current CAM use for diabetes was also greater in ND interested patients.

**Conclusions:**

Patients with sub-optimally controlled type 2 diabetes expressed a high level of interest in trying ND care. Those patients with the greatest interest were less satisfied with their diabetes care, more motivated to engage in self-care, and more likely to use other CAM therapies for their diabetes.

## Background

Risk factor control is generally poor for people in the United States with type 2 diabetes. Estimates from large national surveys, US medical centers and primary care practices all suggest less than half of patients meet recommended targets for the individual cardiometabolic risk factors hemoglobin A1c (HbA1c), blood pressure and lipids, and at most 10% have all three risk factors within recommended target ranges [1-3]. Because risk factor control is directly associated with clinical outcomes and health care costs, strategies to improve risk factor control in patients with diabetes are essential for improving patient health and curbing rising diabetes-related healthcare costs [4,5]. Furthermore, the growing evidence for an association between patient satisfaction with treatment and the treatment experience and improved clinical outcomes [6] suggests that more patient-centered treatment approaches may contribute to risk factor reduction.

Interest in complementary and alternative medical (CAM) treatments has increased over time, and CAM therapies are used by significant numbers of patients with type 2 diabetes. Excluding solitary prayer, estimates of CAM use *by patients with diabetes *range from 39-72% [7-9]. CAM use has been associated with both increased likelihood of receiving preventive care services and increased utilization of primary care, suggesting diabetes patients who use CAM may be especially motivated and engaged in their own health care [10]. However, we could identify only four studies performed in the United States that examined the use of CAM *specifically for diabetes*, two of which also examined associations between the use of CAM and self-care. A national survey by Yeh et al. found that 20% of people with diabetes (n = 95) use CAM, mostly commercial diets and herbal natural products [9]. In an analysis of data from the 2002 National Health Interview Survey (NHIS), Bell and colleagues reported over 72% of people with diabetes use CAM therapies, a higher estimate for CAM use than in people without diabetes (i.e., 61%), though most CAM use *for their diabetes *consisted of diet-based therapies [7]. A small mixed-methods study of 80 people with diabetes from diverse ethnic backgrounds was conducted in three health clinics and a senior center and reported CAM use varied between 15-50% [11]. Finally, a survey of 679 rural North Carolinians with diabetes found that less than 13% used CAM for their diabetes but that healthful eating plans were followed more frequently by CAM users, suggesting again that CAM use and other self-care behaviors may be coupled [12].

Little is known about the effectiveness of multi-modality "whole-system" CAM approaches to treat diabetes. One small, randomized clinical trial of multi-modality, whole-system Ayurvedic care for type 2 diabetes suggested Ayurvedic approaches reduce clinical risk factors, including fasting glucose and hemoglobin A1c [13]. Naturopathic (ND) medicine is defined as CAM by the National Institutes of Health. ND clinical care is of particular interest because it closely mimics primary care in scope of practice with a routine emphasis on intensive lifestyle counseling. Research examining the value of ND care for diabetes is limited to a retrospective analysis of data from patients seeking care from an ND academic clinic. That study found that intensive lifestyle change counseling occurred routinely and improvements in key risk factors, including HbA1c, triglycerides and blood pressure, were achieved concurrently [14]. Because effective methods of delivering lifestyle change counseling in clinical practice are limited, improved access to ND care could provide a useful option for patients with, or at risk for, type 2 diabetes.

In order to determine which types of CAM are being used by patients *for their diabetes *and to determine patients' interest in seeing licensed ND providers (in preparation for a clinical trial) we conducted a telephone survey in members of a large integrated care delivery system (Group Health Cooperative, Seattle, WA). We also examined whether or not the patients most enthusiastic about trying ND care differed from those with less enthusiasm in their current self-care behaviors, perceptions of blood sugar control, motivation to change their self-care, and/or satisfaction with their current health care.

## Methods

### Study Design

Our goal was to administer a telephone-based survey to 200 patients with moderately to poorly controlled type 2 diabetes who receive care from Group Health Cooperative (GHC), a large non-profit, integrated health care system in Washington State. We used electronic medical records to identify potentially eligible patients. All elements of the study were reviewed and approved by the GHC Institutional Review Board.

### Sample

Eligible participants were GHC members aged 21-65 years diagnosed with type 2 diabetes. Because our survey was performed in preparation for a clinical trial, and we were interested in surveying patients with the greatest potential for improvements in clinical risk factors, we only surveyed patients who had a HbA1c value between 7.5-9.5% within the past year in their electronic medical record who also had at least one additional cardiometabolic risk factor (i.e., elevated lipids, blood pressure or obesity). Similarly, we applied the same exclusion criteria in our survey that we planned to apply in our clinical trial. We therefore excluded patients using insulin, those who had a myocardial infarction or stroke within the past six months, and those with a recent history of bariatric surgery or diagnosis of severe psychiatric illness. To avoid interference with other studies, we sampled patients receiving care from clinics outside of the immediate Seattle area. We mailed invitation letters to 350 randomly selected patients meeting our inclusion/exclusion criteria. The letters included a $2 bill and a response card allowing patients to refuse the survey.

### Study Questionnaire

Between 2009-2010, a research specialist telephoned non-refusing patients, explained the study and confirmed eligibility. Once eligibility was confirmed, patients were queried about their demographics (e.g., age, gender, education, ethnicity and income), personal health behaviors, i.e., smoking status; medical history including: year of diabetes diagnosis, history of cardiovascular disease (i.e., heart failure, heart attack and/or stroke); and the presence or absence of micro-vascular complications (i.e., neuropathy, retinopathy, cataracts, and/or nephropathy).

Patients were then asked to rate their health care overall and their health care specifically for their diabetes on 0-10 Likert scales with 0 = "Worst care possible" and 10 = "Best care possible". They were also asked to rate their knowledge of ND care (1-5 Likert scale ranging from 1 = "No knowledge" to 5 = "A lot of knowledge"), whether they had ever visited a ND provider for their diabetes, and about their interest in pursuing ND care *if covered by their health care plan *(1-5 Likert scale ranging from 1 = "Very unlikely" to 5 = "Very likely").

To assess current self-care behavior for diabetes, we used the Summary of Diabetes Self-Care Activities (SDSCA) instrument [15], which captures the number of days in the past week patients engaged in a variety of important self-care activities (e.g., regular physical activity, eating fruits and vegetables, and taking medications as recommended). In order to assess patients' perceptions of the importance of blood glucose control we piloted the Perceptions about Blood Sugar Control (PBSC) instrument. The PBSC is a four question instrument based on the Self-Efficacy Scale [16,17] that uses a 9-point Likert scale ranging from 0 ("Not at all") to 8 ("Extremely") to assess perceptions regarding four domains of glucose control: importance, confidence in self-control of glucose, helpfulness of health care for glucose control, and current status of glucose control. We measured motivation for changing self-care with the Readiness Index (RI) [18], a nine-question instrument that uses a Likert scale ranging from 1 ("Strongly disagree") to 6 ("Strongly agree") to assess three primary domains: evaluation of lifestyle, creating strategies for change and goal commitment. Finally, we adapted questions from the 2007 NHIS [19] to ask patients about their use of specific CAM therapies *for their diabetes *over the past twelve months. Therapies included: vitamin and mineral supplements, body-based therapies such as massage and chiropractic, specific dietary approaches including vegetarian diets, and mind-body therapies like Tai Chi and deep breathing exercises.

### Statistical Analysis

Data were recorded in a master database and analyzed using SAS statistical software version 9.2 (SAS Institute, Cary, NC). Our data analysis plan was developed before the analyses were conducted. Because our primary study questions related to possible differences in self-care, perceptions and readiness to change in ND interested patients vs. patients with less interest we defined "ND Interested" as those patients who said they were "Very likely" to try ND care if covered by their health plan; all other patients were defined as "Less ND Interested". Descriptive statistics were calculated for all patient demographics and health history variables for the entire cohort and then responses were compared using chi^2 ^tests for proportions and t-tests for means to see if there were differences by level of interest in ND care. Our primary analysis compared mean responses to the SDSCA, PBSC and the RI. Finally, the proportions of ND Interested and Less ND Interested patients with history of CAM use were compared for each category of CAM using chi^2 ^tests.

## Results

Of the 350 patients who were sent invitation letters, 56 (16%) refused participation, 45 (13%) could not be contacted, and 29 (9%) either volunteered previously unrecorded medical history requiring their exclusion (including cancer, type 1 diabetes or bariatric surgery) or were no longer current GHC enrollees, leaving 321 eligible patients for inclusion in the sample. Of this remaining sample, 219 patients were successfully interviewed (68.5% of the 321 eligible patients).

Reflecting the demographic characteristics of western Washington, survey respondents had relatively high levels of education and income (Table 1). The mean age of the participants was 55 years and the mean duration of diabetes was 8 years. About two-thirds of respondents were white/non-Hispanic. Average hemoglobin A1c (HbA1c) was 8.1 +/- 0.8%. Over 70% of survey participants had concurrent obesity, hypertension and/or hyperlipidemia, contributing additional cardiovascular disease risk. History of significant cardiovascular disease or micro-vascular complications was rare, although 22% of respondents reported eye/vision-related complications.

**Table 1:** Demographics and Clinical Status: Overall and Compared by ND Interest Subgroups

Demographics and Clinical Status	Total Sample (n = 219)	ND Interested n = 106 (48%)	Less ND Interested n = 114 (52%)	P value
Gender- (Male, N,%)	123 (56%)	59 (56%)	64 (57%)	0.88

Age (Years, Mean, SD)	54.5 (7.9)	54.5 (7.3)	54.5 (8.4)	0.96

Highest Education (N, %)	42 (19%)	21 (20%)	21 (19%)	0.10
High school, GED or less	103 (47%)	57 (54%)	46 (47%)	
Some college, incl. technical College graduate	73 (33%)	57 (26%)	46 (33%)	

Ethnicity (N, %)				
White/Non-Hispanic	149 (68%)	71 (67%)	78 (69%)	0.75

Annual Family Income (N, %)				
< $60,000	73 (33%)	34 (34%)	39 (38%)	0.63
$60,000-80,000	60 (27%)	32 (32%)	28 (27%)	
> $80,000	68 (31%)	33 (33%)	35 (34%)	

Smoking History- Yes (N, %)	29 (14%)	11 (10%)	15 (13%)	0.51

Years of Diabetes (Mean, SD)	8.1(6.0)	7.7(5.2)	8.5(6.7)	0.30

Glycemic Status				
Mean Hemoglobin A1c	8.06 (0.79)	7.97 (0.69)	8.14 (0.87)	0.11

Medical History (N, %)				
Obesity	156 (71%)	72 (68%)	84 (74%)	0.50
Hyperlipidemia	96 (43.8%)	49 (46.2%)	47 (41.6%)	0.49
Hypertension	134 (61.2%)	61 (57.6%)	73 (64.6%)	0.29
Retinopathy/Cataracts	49 (22.5%)	24 (23%)	25 (22%)	0.93
Nephropathy/Renal Disease	28 (13%)	13 (12%)	15 (13%)	0.85
Neuropathy	28(13.%)	13(13%)	15(13%)	0.85
CVD	19 (9%)	11 (10%)	8 (7%)	0.39

Healthcare Rating				
Overall (Mean, SD)	7.8 (1.7)	7.6 (1.8)	7.9 (1.7)	0.26
For Diabetes (Mean, SD)	7.6 (1.9)	7.2 (2.0)	8.0 (1.9)	0.005

Experience & Knowledge of ND Care				
Seen ND Previously (N, %)	21 (10%)	14 (13%)	7 (6%)	0.08
Seen ND for Diabetes (N, %)	9 (4%)	9 (8%)	0 (0%)	0.01
Previous Knowledge of ND Care (N, %)	21 (10%)	15 (14%)	6 (5%)	0.03

Table 1 details the baseline demographics and clinical characteristics of the entire survey sample and compared by ND Interest subgroups. The reported P-values are for differences in proportions compared between subgroups by chi^2 ^test.

Almost half of the patients (48%) said they were "Very likely" to try ND care for their diabetes if covered by their health plan (Table 1). There were no significant differences in demographic or clinical characteristics between subgroups defined by ND interest. Overall, 90% of patients reported very limited knowledge about ND care. However, patients most interested in trying ND care for their diabetes were more likely to have seen a ND for their diabetes in the past (P = 0.01), and to have higher levels of knowledge about ND care (P = 0.03). Although the level of patients' interest in trying ND care was not associated with ratings of their healthcare overall (P = 0.26), ND-interested patients rated their diabetes-specific healthcare significantly lower than those with less interest (P = 0.005).

**Figure 1:**
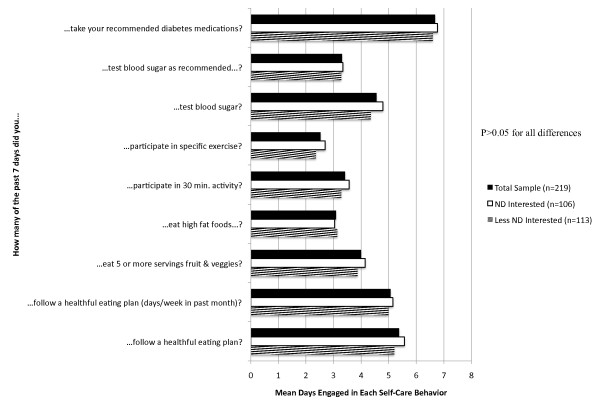
**Summary of diabetes self-care activities: overall and compared by ND interest subgroups**. Figure 1 demonstrates the mean number of days per week survey respondents engaged in specific self-care behaviors. The reported P values are for comparisons of the mean differences between ND Interest subgroups by two-sided t-test. All P-values are > 0.05 unless otherwise noted.

According to their SDSCA responses, participants reported relatively high levels of adherence to taking their medications and following healthful eating plans (Figure 1). Adherence was substantially lower for testing blood sugar as recommended and for participation in regular exercise. Self-care behaviors did not differ significantly by ND interest subgroups (P > 0.05 for each comparison), suggesting ND-interested patients are not currently engaged in more intensive self-care than less interested patients, and therefore have similar room for improvement in their self-care activities.

**Figure 2:**
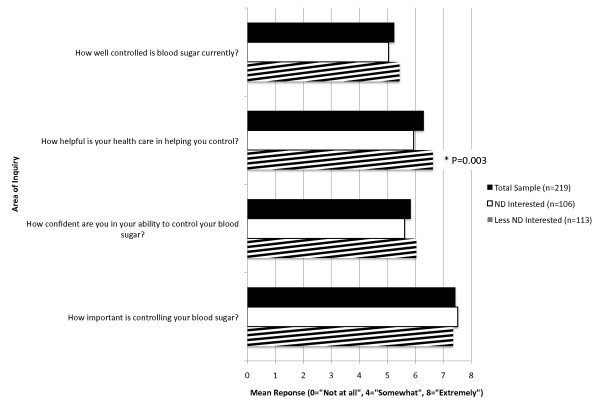
**Perceptions about blood sugar control: overall and compared by ND interest subgroups**. Figure 2 demonstrates the mean response to each question in the Perceptions about Blood Sugar Control instrument based on a 1-8 Likert scale with 0 = "Not at all", 4 = "Somewhat", 8 = "Extremely". All P-values are > 0.05 unless otherwise noted. The reported P-value is for the comparison of the difference in means between ND Interest subgroups by unpaired, two-sided t-test.

Most patients (71%) reported that controlling blood sugar was "Extremely" important, and perceived room for improvement in controlling their blood sugar and in improving their confidence in their ability to do so (Figure 2). Patients' perceptions of the helpfulness of their healthcare in controlling their blood sugar differed significantly by ND interest subgroups, with ND Interested patients perceiving their current health care as less helpful (mean response = 5.9 +/- 1.9 vs. 6.6 +/- 1.5, P = 0.003). All other responses regarding patients' perceptions of blood sugar control were similar between ND interest subgroups.

Respondents generally reported high motivation to change self-care. 59% reported they "Strongly agreed" that they think about the consequences of not changing their self-care and 52% "Strongly agreed" they need to change current self-care behavior. However, only 32% reported that they "Strongly agree" to active planning for changes in their self-care behaviors. Although respondents were generally highly motivated to change self-care (Table 2), responses by ND-interested patients were significantly higher in several key motivational areas including: having a plan for changing self-care (P = 0.01), determination to succeed in making self-care changes (P = 0.007), and commitment to making lasting changes in self-care (P = 0.02).

**Table 2:** Readiness Index: Overall and Compared by ND Interest Subgroups

Area of Readiness Index	Total Sample (n = 219) Mean (SD)	ND Interested (n = 106) Mean (SD)	Less ND Interested (n = 114) Mean (SD)	P value
1. Think about consequences of not changing SC:	5.28 (1.05)	5.41 (0.91)	5.16 (1.15)	0.08

2. Feel current SC could be improved	4.36 (1.44)	4.49 (1.31)	4.23 (1.54)	0.18

3. Need to change SC:	5.02 (1.21)	5.02 (1.23)	5.03 (1.2)	0.96

4. Have plan to change SC:	4.59 (1.24)	4.81 (1.16)	4.38 (1.28)	0.01

5. Think about fitting SC into life:	4.92 (1.06)	5.01 (1.06)	4.83 (1.06)	0.22

6. Have plan to overcome SC barriers:	4.11 (1.30)	4.14 (1.32)	4.09 (1.29)	0.77

7. Willing to make sacrifices for SC:	4.89 (1.00)	5.01 (0.98)	4.77 (1.01)	0.08

8. Determined to succeed in SC:	4.96 (1.00)	5.15 (0.90)	4.79 (1.06)	0.007

9. Committed to making lasting changes in SC:	4.89 (1.00)	5.60 (0.94)	4.73 (1.03)	0.02

Table 2 details mean responses to the Readiness Index instrument by the entire survey sample and compared by ND Interest subgroups. Mean responses are based on a 1-6 Likert scale with 1 = "Strongly disagree", 4 = "Agree", 6 = "Strongly agree". The reported P-values are for differences in means between ND Interest subgroups compared by two-sided t-test. SC = self-care.

The CAM therapies most commonly used for diabetes were natural products, mind-body therapies, and diet-based therapies, which were used by 43%, 23% and 17% of the total survey sample respectively (Table 3). Respondents in the ND Interested subgroup were significantly more likely to use Natural Products (56% vs. 30%, P = 0.0001) and Mind-Body Therapies (32% vs. 14%, P = 0.002).

**Table 3:** CAM Therapies Used Specifically for Diabetes: Overall and Compared by ND Interest

General Category of CAM Therapy (N, %)	Total Sample (n = 219)	ND Interested (n = 106)	Less ND Interested (n = 114)	P value
Natural Products	93 (43%)	59 (56%)	34 (30%)	0.0001

Mind-Body Therapies	50 (23%)	34 (32%)	16 (14'%)	0.002

Manipulative/Body-based Therapies	20 (9%)	7 (7%)	13 (12%)	0.21

Whole Medical Systems	12 (6%)	9 (8%)	3 (3%)	0.06

Diet-based Therapies	37 (17%)	23 (22%)	14 (12%)	0.07

				

**Individual CAM Therapy** **(N, %)**	Total Sample (n = 219)	ND Interested (n = 106)	Less ND Interested (n = 114)	P value

Vitamin/mineral supplements	73 (34%)	49 (46%)	24 (22%)	0.0001

Herbal/nutritional supplements	51 (24%)	39 (38%)	12 (11%)	0.0001

Meditation	32 (15%)	22 (21%)	10 (9%)	0.01

Deep breathing exercises	34 (16%)	23 (22%)	11 (10%)	0.05

Massage therapy	14 (6%)	5 (5%)	9 (8%)	0.33

Vegetarian diet	14 (6%)	9 (8%)	5 (2%)	0.22

Yoga	12 (5%)	7 (7%)	5 (2%)	0.48

Low glycemic index diet	12 (5%)	7 (7%)	5 (2%)	0.48

High-dose vitamin/mega-vitamins therapy	12 (5%)	9 (8%)	3 (3%)	0.06

Chiropractic or Osteopathic Manipulation	9 (4%)	5 (5%)	4 (4%)	0.66

Progressive Muscle Relaxation	9 (4%)	6 (6%)	3 (3%)	0.26

South Beach diet	9 (4%)	5 (5%)	4 (4%)	0.66

Atkins diet	7 (3%)	4 (4%)	3 (3%)	0.64

Folk medicine	5 (2%)	3 (3%)	2 (2%)	0.60

Table 3 reports the frequency of CAM use by the entire survey sample and compared by ND Interest subgroups. The reported P-values are for differences in proportions compared between subgroups by chi^2 ^test.

## Discussion

Our results suggest that use of and interest in complementary and alternative medicine (CAM) is high among patients with type 2 diabetes. Current and previous CAM use and knowledge of ND care were associated with greater interest in ND services *for diabetes*. Our findings also suggest that patients' interest in CAM may partly reflect dissatisfaction with their current diabetes care, and not solely an increased inclination to participate in self-care behaviors. In fact, respondents with greater interest in ND services reported similar levels of all self-care behaviors.

These findings differ from those of the ELDER study which found significant associations between use of CAM for diabetes and increased self-care behaviors, including more days following a healthful eating plan, monitoring glucose, engaging in foot care and adhering to medication [12]. These divergent findings may be explained by differences in the patient populations surveyed and the analyses performed. Our study focused on patients who were not in optimal glycemic control while the ELDER study included patients with a broad range of risk factor control. As a result, the baseline HbA1c was higher in our sample than the ELDER sample (mean HbA1c: 8.1% vs. 6.8%, respectively). The narrower distribution of glycemic control in our cohort may be associated with a correspondingly narrower range of self-care behavior, thus making it more difficult to identify distinct subgroups. Because self-management for diabetes tends to be positively associated with patient education and health literacy [20-22], the ELDER study's greater variation in educational status may have led to lower mean estimates of self-care and a greater division between CAM users and non-users. Finally, our statistical comparisons were performed based on the mean number of days engaged in each behavior, whereas Bell et al. compared the proportion above specified thresholds in frequency of each behavior.

Patients in our study using more CAM practices and expressing more interest in ND care, reported higher motivation toward self-care. However, patients with greater interest in ND care rated care for their diabetes *lower *than patients in the less interested subgroup, despite similar ratings of their health care overall. ND interested patients also consistently scored their health care as *less helpful *for *controlling *their blood glucose, whereas all other measures of the importance of blood glucose control were equivalent for the two groups. Collectively, these findings suggest attitudes surrounding diabetes-specific health care services combined with increased motivation, rather than increased personal engagement in self-care, may mediate interest in CAM and ND care.

The findings from this survey have important clinical implications for conventional and CAM clinical care settings. In general, our sample had a high frequency of elevated cardiometabolic risk factors, and therefore risk reductions and subsequent improvements in clinical outcomes are possible if these patients were to be matched with effective services, perhaps including the one-on-one dietary and physical activity counseling recommended per preventive service guidelines [23]. For CAM clinical care settings, our findings that CAM interest is high amongst this patient population and that CAM-interested patients are motivated to make lasting changes in their self-care, suggests an important responsibility of CAM providers to assist these patients in reaching their self-care goals. For conventional care settings, our findings suggest CAM interest by patients may be due, at least in part, to dissatisfaction with their current diabetes care. Because these patients are also motivated to change, it is important to either engage them in their current care or direct them to services where they can channel their motivation. Finally, we found 43% of patients used natural products for their diabetes care, an estimate that exceeds those published elsewhere. Given the potential for medication/natural product interactions [24], this high prevalence of concurrent use should encourage dialogue between patients, physicians, pharmacists, and CAM providers to ensure patient safety.

The major limitation of this survey is its restriction to members of a single health plan in Washington State. Because our sample was nearly 70% white and nearly 80% had at least some college education, our results may not apply to geographic regions with more diverse populations. In particular, because CAM use has been associated with higher levels of education in other observational studies [25], CAM use in our highly educated population may be greater than that in other populations.

A high priority for diabetes research is how best to design, implement and evaluate innovative programs in primary care that motivate patients to adopt self-care behaviors [26]. Because ND practice emphasizes intensive lifestyle counseling [14], this model of care has the potential to improve self-care behaviors of persons with diabetes and reduce the risks of undesirable outcomes. The actual effect of ND care on clinical outcomes should be the focus of future trials. In the meantime, recognition that CAM interest may represent both increased patient motivation and dissatisfaction with current care may help all providers align patients with the necessary resources to facilitate improvements in their self-care.

## Conclusions

We found that patients interested in naturopathic (ND) care did not report higher levels of self-care behavior, which contrasts the common belief that users of CAM therapies are inherently more engaged in their self-care. We did find, however, that ND Interested patients were more highly motivated to change their behaviors than those with less interest. Another novel finding of this study has been that ND interested patients rated their diabetes care less favorably and considered healthcare less helpful for glucose control, feelings which may contribute to their greater interest in trying alternative approaches to their diabetes care. Our results document, possibly for the first time, a significant use of CAM therapies, especially natural products, specifically *for diabetes*.

The totality of these findings suggest there is a subgroup of patients with type 2 diabetes who are not satisfied with their current diabetes care, but are highly motivated to improve their self-care behaviors by trying new approaches, such as CAM therapies and ND care. These circumstances suggest that the time is right to conduct scientifically rigorous studies evaluating the effectiveness and cost-effectiveness of ND care and perhaps other CAM therapies for this common, debilitating and costly health problem.

## Competing interests

The authors declare that they have no competing interests.

## Authors' contributions

Authors contributed to this manuscript in the following ways: RB and DC wrote the manuscript; LG and JL evaluated the data and wrote the statistical analysis section; and KS, SC, and CC edited and reviewed the manuscript. All authors read and approved the final manuscript.

## Pre-publication history

The pre-publication history for this paper can be accessed here:

http://www.biomedcentral.com/1472-6882/11/121/prepub
